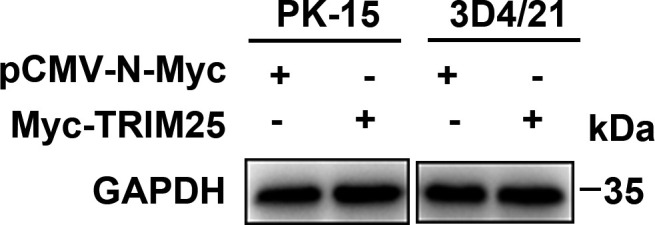# Correction for Wu et al., “CSFV restricts necroptosis to sustain infection by inducing autophagy/mitophagy-targeted degradation of RIPK3”

**DOI:** 10.1128/spectrum.03188-24

**Published:** 2025-01-28

**Authors:** Keke Wu, Bingke Li, Xiaoai Zhang, Yiqi Fang, Sen Zeng, Wenshuo Hu, Xiaodi Liu, Xueyi Liu, Zhimin Lu, Xiaowen Li, Wenxian Chen, Yuwei Qin, Bolun Zhou, Linke Zou, Feifan Zhao, Lin Yi, Mingqiu Zhao, Shuangqi Fan, Jinding Chen

## AUTHOR CORRECTION

Volume 12, no. 1, e02758-23, 2024, https://doi.org/10.1128/spectrum.02758-23. Page 16, Fig. 7A: The GAPDH panels should appear as shown in this correction. Regretfully we inadvertently repeated the cropping of images, resulting in a duplication of loading control bands. The current values, though, are correct and the conclusions remain intact. We apologize for this error, which did not change the final result.

**Fig 7:**