# Supplementary material for: CSFV restricts necroptosis to sustain infection by inducing autophagy/mitophagy-targeted degradation of RIPK3
Source: Microbiol Spectr. 2023 Dec 15;12(1):e02758-23. doi: 10.1128/spectrum.02758-23 (PMC10782971; doi:10.1128/spectrum.02758-23)
Supplement: Supplemental material — Figures S1 to S5. [file spectrum.02758-23-s0001.pdf]

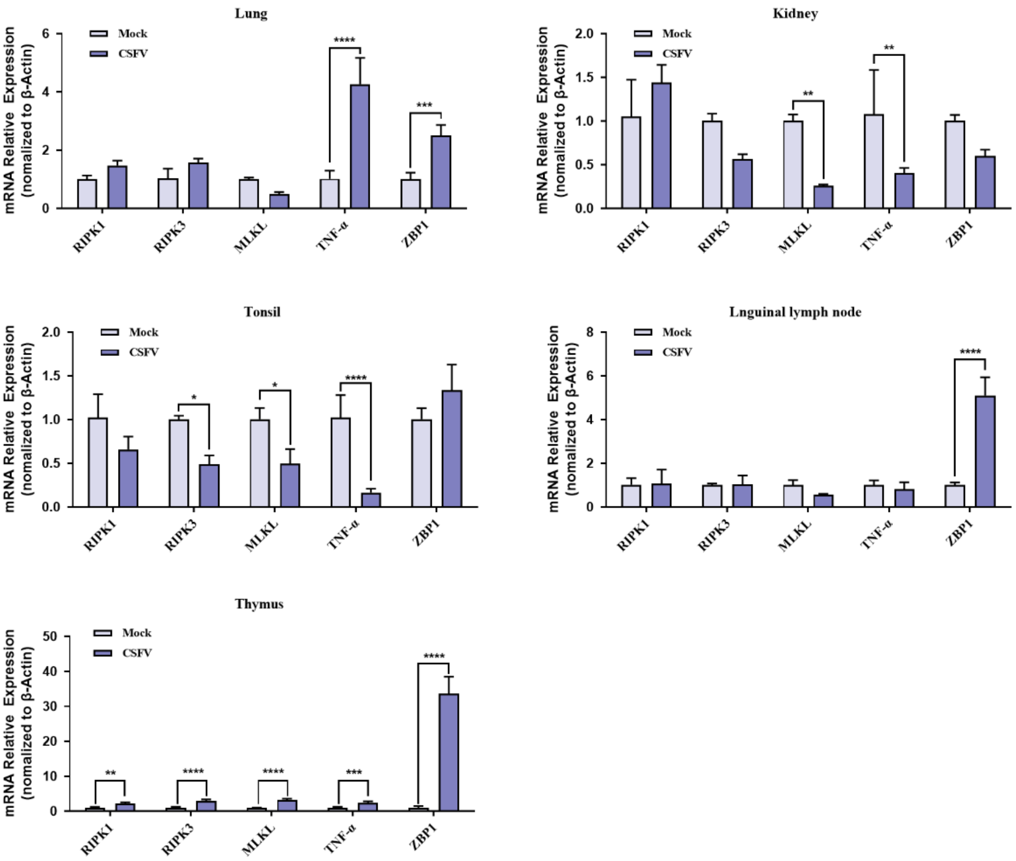

Figure S1. Differential regulation of necroptosis in different tissues by CSFV infection. qRT-PCR was used to determine the mRNA levels of necroptosis marker genes including *RIPK1*, *RIPK3*, *MLKL*, *TNF-α* and *ZBP1* in lung, kidney, tonsil, lymph node and thymus tissues from CSFV infected and uninfected piglets at 7 d. The fold changes were related to the internal control β-ACTIN. Error bars indicate the mean (±SD) of 3 independent experiments. *, $P < 0.05$, **, $P < 0.01$, ***, $P < 0.001$ and ****, $P < 0.0001$(two-way ANOVA).

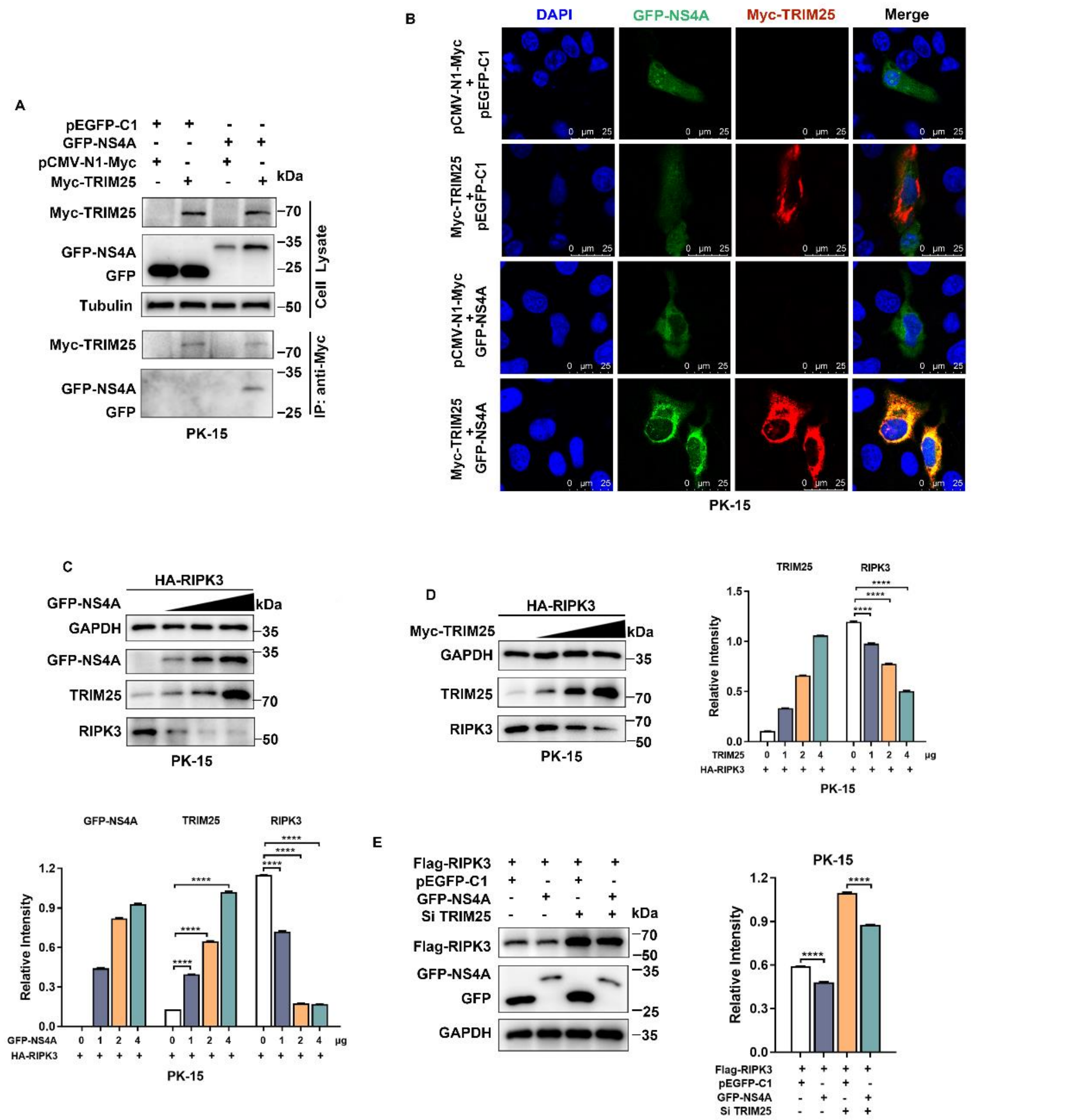

Figure S2. CSFV NS4A interacts with TRIM25. (A) Myc-TRIM25 was co transfected with pEGFP-C1 and GFP-NS4A in PK-15 cells, while a control group of cells were set up in which pCMV-N1-Myc was co-transfected with pEGFP-C1 and GFP-NS4A, cells were lysed, Co-IP experiments, and Western blot analysis was performed. (B) Myc-TRIM25 was co transfected with pEGFP-C1 and GFP-NS4A in PK-15 cells, while a control group of cells were set up in which pCMV-N1-Myc was co-transfected with pEGFP-C1 and GFP-NS4A, fixed cells were incubated with anti-Myc tag primary antibody and then stained with Alexa fluor 555 conjugated anti-Rabbit-IgG secondary antibody (red) and nuclei were stained with DAPI. Scale bars: 25 µm. (C) HA-RIPK3 was co transfected with increasing amounts of GFP-NS4A (wedge-

shaped, 0, 1, 2, 4 μg) into PK-15 cells for 24 h, and the cell lysates were detected by Western blot. The Gray scale value analysis was related to the internal control GAPDH (lower panel). Error bars indicate the mean (±SD) of 3 independent experiments. ****, $P < 0.0001$ (two-way ANOVA). (D) HA-RIPK3 was co transfected with increasing amounts of Myc-TRIM25 (wedge-shaped, 0, 1, 2, 4 μg) into PK-15 cells for 24 h, and the cell lysates were detected by Western blot. The Gray scale value analysis was related to the internal control GAPDH (right panel). Error bars indicate the mean (±SD) of 3 independent experiments. ****, $P < 0.0001$ (two-way ANOVA). (E) Si TRIM25 was co transfected with pEGFP-C1 and GFP-NS4A in PK-15 cells, while a control group of cells were set up in which Si NC was co-transfected with pEGFP-C1 and GFP-NS4A, the cell lysates were detected by Western blot. The Gray scale value analysis was related to the internal control GAPDH (right panel). Error bars indicate the mean (±SD) of 3 independent experiments. ****, $P < 0.0001$ (two-way ANOVA).

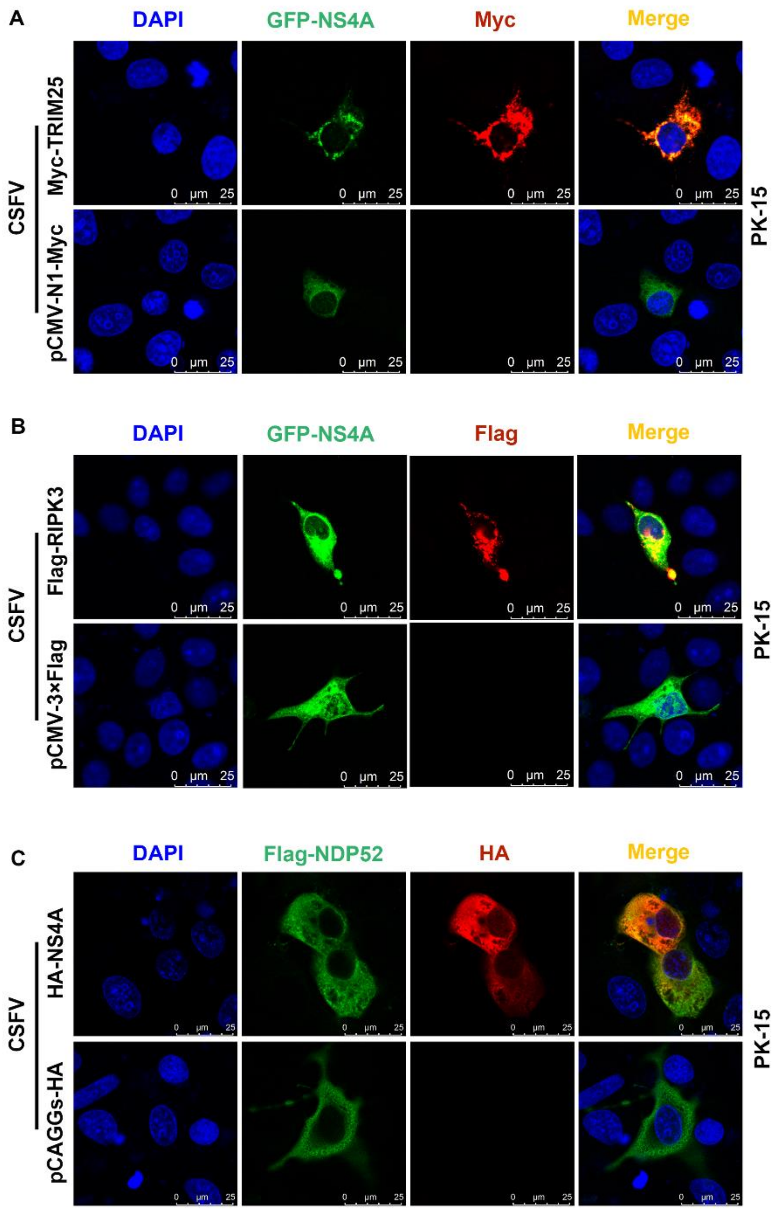

Figure S3. Co-localization of NS4A with its interacting proteins during CSFV infection. (A) and (B) GFP-NS4A and Myc-TRIM25 (A)/Flag-RIPK3 (B) were co-transfected into PK-15 cells. Subsequently, the cells were infected with CSFV(MOI=1). As a control group, cells were transfected with pCMV-N1-Myc or pCMV-3×Flag. After transfection and infection, the cells were fixed and incubated with anti-Myc antibody or anti-Flag antibody. Subsequently, the cells were incubated with Alexa Fluor 555-conjugated anti-rabbit IgG secondary antibody (red) and stained with DAPI. Scale bars: 25 μm. (C) HA-NS4A and Flag-NDP52 were co-transfected into PK-15 cells.

Subsequently, the cells were infected with CSFV(MOI=1). As a control group, cells were transfected with pCAGGs-HA. The fixed cells were incubated with anti-Flag primary antibody and anti-HA primary antibody, followed by Alexa fluor 488 conjugated anti-Mouse-IgG secondary antibody (green) and Alexa fluor 555 conjugated anti-Rabbit-IgG secondary antibody (red), and the nuclei were stained with DAPI. Scale bars: 25 μm.

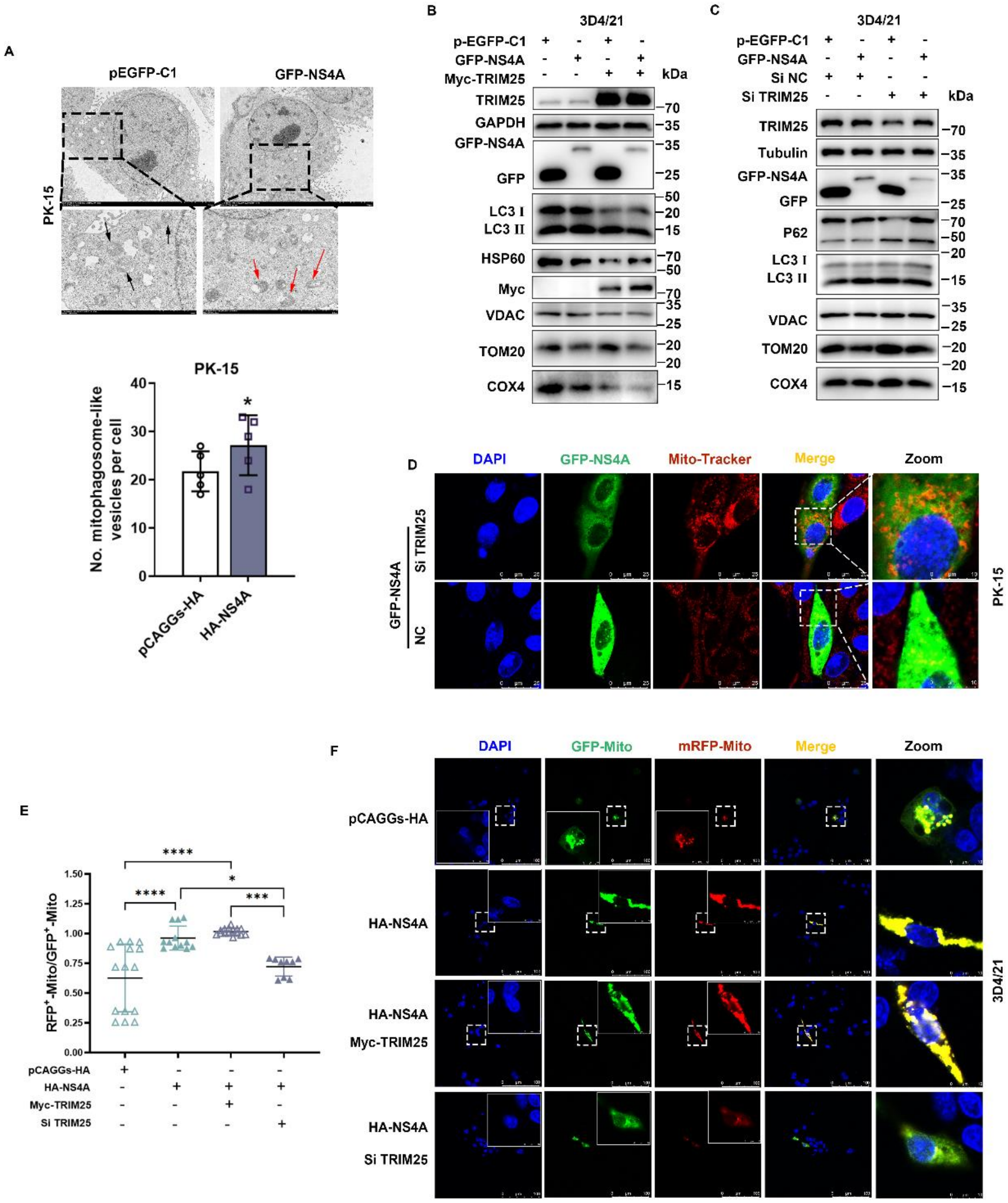

Figure S4 CSFV NS4A synergizes TRIM25 to induce mitophagy. (A) Mitochondrial structures and autophagy like vesicles were visualized by transmission electron microscopy (upper panel), and the number of autophagic vesicles with encapsulated mitochondria was quantified (lower panel). Error bars indicate the mean (±SD) of 5 independent experiments. *, $P < 0.05$ (one-way ANOVA). (B) Myc-TRIM25 was co-transfected with pEGFP-C1 and GFP-NS4A in 3D4/21 cells, respectively, while a control group of cells was set up in which pCMV-N1-Myc was co-transfected with

pEGFP-C1 and GFP-NS4A, respectively. Expression levels of autophagy associated proteins p-mTOR, LC3-II and P62 and mitochondrial associated proteins HSP60, TOMM20, COX IV and VDAC in cell lysis products were detected by Western blot. (C) Si TRIM25 was co-transfected with pEGFP-C1 and GFP-NS4A in 3D4/21 cells, respectively, while a control group of cells was set up in which Si NC was co-transfected with pEGFP-C1 and GFP-NS4A, respectively. Expression levels of autophagy associated proteins p-mTOR, LC3-II and P62 and mitochondrial associated proteins HSP60, TOMM20, COX IV and VDAC in cell lysis products were detected by Western blot. (D) PK-15 cells were co-transfected with GFP-NS4A together with Si TRIM25 for 24 h, while setting up a control group of co-transfected Si NC cells, immunostained with Mito-Tracker, and nuclei were stained with DAPI. Scale bars: 25 and 10 μm. (E) The fluorescence intensity of GFP-Mito (green) and RFP-Mito (red) of Figure 7J. (F) HA-NS4A was co-transfected with Myc-TRIM25 and Si TRIM25 into 3D4/21 cells, respectively, and a cell control transfected with HA-NS4A only and pCAGGs-HA only were set up, along with mitophagy dual fluorescent reporter plasmid Mito-mRFP-EGFP to visualize the mitochondria-lysosome delivery (upper panel).

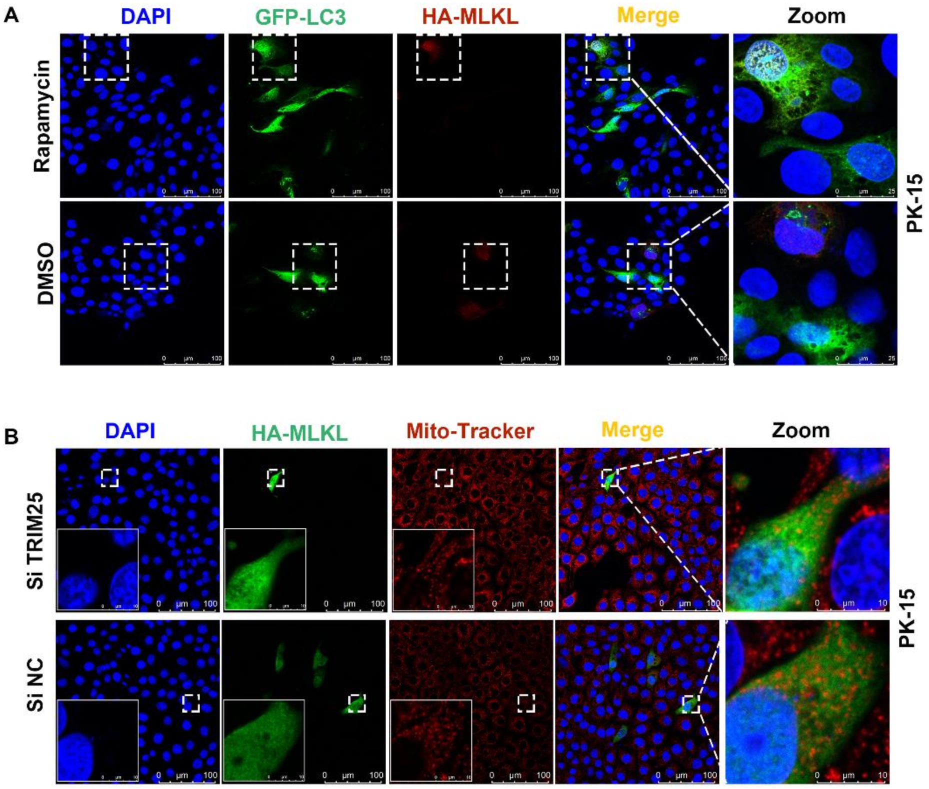

Figure S5. MLKL has no co-location with LC3 or mitochondria. (A) HA-MLKL and GFP-LC3 were co-transfected into PK-15 cells for 24 h, and then treated with rapamycin (100 nM) for 6 h, while setting up a control group of cells treated with DMSO. The cells were incubated with anti-HA primary antibody, followed by Alexa fluor 555 conjugated anti-Rabbit-IgG secondary antibody (red), and the nuclei were stained with DAPI. Scale bars: 100 and 25 μm. (B) PK-15 cells were co-transfected with HA-MLKL together with Si TRIM25 for 24 h, while setting up a control group of co-transfected Si NC cells, the cells were immunostained with Mito-Tracker, incubated with anti-HA primary antibody, followed by Alexa fluor 488 conjugated anti-Mouse-IgG secondary antibody (green), and nuclei were stained with DAPI. Scale bars: 100 and 10 μm.
